# Prevention and alleviation of allergic rhinitis by oral administration of *Lacticaseibacillus paracasei* GOLDGUT-Lpc969

**DOI:** 10.3389/fimmu.2024.1444778

**Published:** 2024-09-30

**Authors:** Xiaoli Zhou, Xizi Song, Ting Shu, Silu Zhang, Zhizhu Zhang, Canying Hu, Jie Pan, Xiaoshuang Dai, Huaijie Hao, Guoxun Xiao, Pengfei Wang, Kai Liu

**Affiliations:** ^1^ Wonderlab Innovation Centre for Healthcare, Shenzhen Porshealth Bioengineering Co., Ltd, Shenzhen, China; ^2^ Department of Research and Development, Shenzhen Xbiome Biotech Co., Ltd., Shenzhen, China; ^3^ Emergency Department, Sun Yat-sen Memorial Hospital, Guangzhou, China; ^4^ Department of Rehabilitation Medicine, University of Health and Rehabilitation Sciences Qingdao Hospital (Qingdao Municipal Hospital), Qingdao, China

**Keywords:** allergic rhinitis, *Lacticaseibacillus paracasei*, Th2 immune response, probiotic, immunomodulation, gene K03671

## Abstract

**Introduction:**

Allergic rhinitis (AR) is a widespread upper airway disorder characterized by inflammation of the nasal passages. It is immunologically mediated via the hypersensitivity type I mechanism, which is primarily elicited by the immunoglobulin E (IgE)-linking allergen-induced imbalance of the Th2/Th1 immune response. Owing to the limited efficacy of current medications, probiotics have received attention for their potential in preventing and ameliorating AR.

**Methods:**

In this study, a *Lacticaseibacillus* paracasei strain, GOLDGUTLpc969 (Lpc969), isolated from the feces of healthy adults, was proven to be effective in preventing AR by LPA-induced RBL-2H3 *in-vitro* and OVA-induced AR mice *in-vivo* evaluation.

**Results:**

The strain significantly attenuated the release of histamine and degranulation in LPS-induced RBL-2H3 cells. In the OVA-induced AR mice, *L. paracase*i GOLDGUT-Lpc969 also exhibited a significant decrease in disease indicators such as the disease activity index (DAI score), serum IgE, and serum histamine. Treatment with *L. paracasei* GOLDGUT-Lpc969 led to significant suppression of the Th2-related cytokines IL-4, IL-5, IL-6, IL-13, and TNF-α in the serum of mice.

**Discussion:**

Furthermore, a comparison of the genomes of three previously reported AR-effective *L. paracasei* strains (including GOLDGUTLpc969) and one non-effective *L. paracasei* strain revealed that the gene K03671 may play a key role in alleviating AR symptoms. In conclusion, this study highlights the efficacy of *L. paracasei* GOLDGUT-Lpc969 in AR prevention by suppressing the Th2 immune response and proposes the potential involvement of the functional gene K03671 in ameliorating AR symptoms. Therefore, *L. paracasei* GOLDGUT-Lpc969 shows promise as a probiotic for preventing AR.

## Introduction

Allergic rhinitis (AR) is an upper-airway disorder characterized by inflammation of the nasal passages, commonly caused by allergic reactions to allergens such as pollen, dust mites, or animal dander. According to the World Allergy Organization, the global prevalence rate of AR ranges from 10% to 30% in adults (1), of which approximately 3.6% of AR patients experience work absenteeism, and 36% experience reduced work productivity (2). However, the current treatments for AR patients include immunotherapy, which is expensive, leukotriene receptor antagonists (LTRAs), and intranasal steroids, which are linked to a higher incidence of side effects and a limited therapeutic benefit (3–6). Thus, there is still a need to create safe and efficient therapeutic and preventive approaches for such public health issues.

During the pathological development of AR, the Th2-predominant response is brought on by various phases of the sensitization process. On the one hand, it leads to the hyperreactivity of the immune system to allergens, prompting the body to generate specific immunoglobulin E (IgE) and to bind to the surface of mast cells in response to the allergens (7). Upon subsequent exposure, the allergen can bind to the IgE on the mast cells, triggering their degranulation and releasing mediators, including immediately released histamine and delayed released leukotrienes (8, 9). These mediators, through multifaceted interactions, ultimately culminate in the manifestation of AR symptoms. On the other hand, the excessive activation of Th2 contributes to the persistence and chronicity of allergic inflammation (10), making targeting Th2 cells and specific IgE a promising therapeutic approach in managing allergic rhinitis.

Recently, a growing body of research in both humans and animals has linked the emergence of allergic disease to modifications in the microbiome (11). For instance, neonatal rhesus macaques with antibiotic-disrupted gut microbiota exhibited a compromised pulmonary immune system, characterized by an increased susceptibility to pneumonia, a hyperinflammatory transcriptional signature, and dysregulated homeostatic pathways within the host (12). Moreover, an analysis of bacterial DNA extracted from fecal samples of 92 asthmatic children and 88 healthy children indicated a reduction in the abundance of *Akkermansia muciniphila* and *Faecalibacterium prausnitzii* in the allergic asthma group compared with the healthy group. These species have been implicated in the suppression of inflammation through the regulation of secreted metabolites, as suggested by an increase in interleukin-10 (IL-10) and a decrease in interleukin-12 (IL-12) levels (13). Similarly, clinical research suggests that with the elevation of inflammatory factors in the peripheral serum of children with asthma, their probability of intestinal flora disturbance and gastrointestinal incommensurate symptoms will be increased (14). These data support the crosstalk between gut bacteria and systemic immunity during homeostasis and allergy, suggesting that the introduction of certain probiotics may be a potential strategy to prevent allergic inflammation in the respiratory system.

Targeting the gut-lung link, numerous probiotics have been investigated for their immunoregulatory effects (15–22). *Lacticaseibacillus*, formerly known as *Lactobacillus*, is a genus from which some species have been shown to have a preventive effect on respiratory allergy. Among *Lacticaseibacillus*, strains of the *Lacticaseibacillus rhamnosus* species have exhibited ameliorative and preventive effects on ovalbumin (OVA)-induced allergic mice, supported by the decreased serum IgE levels, rebalancing of Th1/Th2, and Treg/Th17 responses (23, 24). Additionally, it was uncovered that exopolysaccharides of *L. rhamnosus* strain LOCK900 were found to induce IgA production, which helped to explain the ability of this strain to alleviate airway inflammation (25). Moreover, species such as *Lacticaseibacillus casei* have been mentioned for their potential to attenuate airway disease or regulate immune responses (26, 27). *Lacticaseibacillus paracasei* is another species that has received considerable attention in the study of AR. *L. paracasei* strain MG4272 significantly suppressed the release of Th2-related cytokines such as TNF-α, IL-6, IL-4, IL-5, and IL-13 in RAW 264.7 and RBL-2H3 cells. It also modulated the phosphorylation of STAT6 in macrophages and mast cells (22). Similarly, *L. paracasei* strain GM-080 (LP-33, BCRC-910220, CCTCC M 204012) was reported to have an alleviative effect on OVA-induced mice by oral administration (28). However, in the same species, *L. paracasei* strain BCRC 16100 demonstrated negative feedback in airway allergy (28). In conclusion, probiotics from the genus *Lacticaseibacillus* have been shown to be beneficial in the treatment and prevention of AR, although it is noteworthy that the effect of probiotics varies between individuals; therefore, further research is needed to identify the effective strains, dosages, and duration of treatment.

In this study, *L. paracasei* strain GOLDGUT-Lpc969 (Lpc969), a strain isolated from the feces of healthy adults, was selected as an effective probiotic in the prevention and alleviation of AR by oral administration after a series of *in vitro* and *in vivo* experiments. The strain was first tested for its effect on histamine secretion and degranulation of the RBL-2H3 cell line. Subsequently, the effects of *L. paracasei* GOLDGUT-Lpc969 on OVA-induced allergic mice were evaluated by assessing serum immunoglobulin (Ig) levels, inflammatory cell infiltration, and serum cytokine profiles. In addition, comparative genomic analysis between GOLDGUT-Lpc969 and other AR effective/non-effective strains was investigated to mine the potential functional genes of effective strains in terms of AR prevention and alleviation.

## Materials and methods

### Bacteria, cells, and culture conditions

*L. paracasei* strain GOLDGUT-Lpc969 was isolated using the serial dilution method from the fecal sample collected from a healthy adult donor in Shenzhen, China. The pure colony was picked from a De Man–Rogosa–Sharpe (MRS) agar plate and identified by colony morphologies, such as color, size, shape, transparency, and biochemical characteristics, performed using an API 20A card (BioMérieux) according to the instruction manual. The strain was inoculated and cultured overnight in the MRS medium at 37°C under anaerobic conditions. The bacterial pellet was collected by centrifugation at 4°C and 8,000 g for 10 min and washed once with PBS. The bacterial cells were then resuspended in PBS containing 15% glycerol, aliquoted into cryovials, and stored at −80°C for use in animal experiments.

The RBL-2H3 cell line was purchased from the Chinese National Infrastructure of Cell Line Resource and cultured in minimum essential medium (MEM, Gibco) supplemented with 10% v/v fetal bovine serum (FBS, Solarbio), 1% penicillin, 1% streptomycin, and 1% non-essential amino acids in a humidified atmosphere of 5% CO_2_ at 37°C. Cells were passaged when the cell density exceeded 80% and log phase cells were inoculated into a 24-well plate at 400 μl (2×10^5^ cells) per well for further experimentation.

### Whole-genome sequencing and comparative genomic analysis

The whole genome of strain GOLDGUT-Lpc969 was sequenced using Nanopore PromethION and Illumina NovaSeq from the Novogene Company and the generated reads were assembled using Unicycler (version 0.4.8) to combine PE150 data and Nanopore data. We used GeneMarkS (version 4.17) to retrieve the associated coding gene, RepeatMasker (version 4.0.5) to predict the interspersed repetitive sequences, tRNAscan-SE (version 1.3.1) to predict the transfer RNA, rRNAmmer (version 1.2) to predict the ribosomal RNA (rRNA), phiSpy (version 2.3) to predict the prophage, and CRISPRdigger (version 1.0) to identify the CRISPR. The whole-genome blast search was performed against the KEGG, COG, and GO databases for the prediction of gene functions (29). To access the safety of strain GOLDGUT-Lpc969 *in silico*, the translated amino acid sequences of coding genes were aligned against the Antibiotic Resistance Genes Database (ARDB) (E-value ≤ E-10, identification ≥ 40%) and the Virulence Factor Database (VFDB) (E-value ≤ E-8, with identification ≥ 40%) (30).

The phylogenetic tree was constructed based on the core genomes of strain GOLDGUT-Lpc969 and the related genomes within the same genus. First, the comparative genomic analysis of the genomes was performed using Orthofinder v2.5.4 (31). Then, 3,993 homologous genes harbored by each genome were defined as the core genomes. All genes were aligned using MUSCLE (32), and columns with more than 95% gaps were trimmed using trimAL (33). Maximum likelihood trees of the genomes were constructed using IQ-TREE (34) and UFBoot (35) with a substitution model of LG+F+R10; the number of bootstraps was 100 and the evolutionary mode was LG+GAMMA. Finally, the trees were visualized on the iTOL web server (36).

### IgE-mediated RBL-2H3 degranulation assay

Rat basophilic leukemia (RBL)-2H3 cells were used to investigate the anti-allergic effects of the strain GOLDGUT-Lpc969, as previously described (37). Briefly, RBL-2H3 cells were incubated with 0.2 μg/ml anti-DNP-IgE (Sigma-Aldrich, Shanghai, China) for 16 h under 5% (v/v) CO_2_ at 37°C to sensitize the cells. After the stimulants were washed out, the cells were exposed to different concentrations of strain GOLDGUT-Lpc969 (Lpc969, MOI = 10 or 1) for 3 h; the cells without bacterial exposure were used as a model control. The cells were then stimulated with 1 μg/ml dinitrophenyl coupled to human serum albumin (DNP-HAS, 4ADI) for 15 min. The untreated cells were used as a negative control. The culture supernatants were collected to measure the histamine content and β-hexosaminidase activity as indicators of degranulation. To assess the maximal release of β-hexosaminidase, the stimulated cell pellet was lysed with 0.1% (v/v) Triton X-100 (Sigma), and the lysis solution was collected. In some experiments, the sensitized RBL-2H3 cells were incubated with 20 μg/ml azelastine hydrochloride (AZE, Macklin) and used as a positive control for the strain GOLDGUT-Lpc969-treated groups.

### Detection of histamine content and β-hexosaminidase activity

The levels of histamine and β-hexosaminidase released from RBL-2H3 cells were determined as previously reported (37). To detect histamine levels, 20 μl of 1 M NaOH solution was added to 100 μl of cell culture supernatant, and 25 μl of reaction solution (1% o-phthalaldehyde dissolved in methanol) was immediately added and thoroughly mixed. The reaction was incubated at room temperature for 4 min and then stopped by adding 10 μl of 3M HCl solution. Fluorescence intensity was then measured using a VarioskanTM LUX multimode microplate reader (Thermo Fisher Scientific) with an excitation wavelength of 355 nm and an emission wavelength of 460 nm. The relative amount of histamine for each well was calculated according to the following equation:


$$\begin{array}{c} \text{Relative amount of histamine}\\ ={\text{OD}}_{460}\;\left(\text{sample}\right)/{\text{OD}}_{460}\;\left(\text{average of control}\right) \end{array}$$

OD_460_ (sample) is the fluorescence intensity at 460 nm of the sample tested, and OD_460_ (average of control) is the average fluorescence intensity at 460 nm of the control group.

To detect the amount of β-hexosaminidase, 25 μl of cell culture supernatant was added to an equal volume of substrate solution [5 mM 4-nitrophenyl-N-acetyl-β-glucosaminide (NP-GlcNAc) in 0.2 M sodium citrate buffer, pH 4.5, all purchased from Sinopharm Chemical Reagent Co., Ltd., Shanghai, China] and incubated for 1.5 h at 37°C. The reaction was stopped by the addition of 200 μl of stop solution (0.1 M Na_2_CO_3_/NaHCO_3_, pH 10.0). Fluorescence intensity was measured using a VarioskanTM LUX multimode microplate reader with an excitation wavelength of 355 nm and an emission wavelength of 405 nm. The relative hexosaminidase release was calculated using the following equation:


$$\begin{array}{c} \text{Relative hexosaminidase release }\left(\%\right)\\ ={\text{OD}}_{405}\;\left(\text{sample supernatant}\right)/{\text{OD}}_{405}\;\\ \left(\text{cell pellet lysed with }0.1\%\left(\text{v}/\text{v}\right)\text{Triton X}-100\right)\times 100 \end{array}$$

### Animals

Six-week-old BALB/c mice (18 ± 2 g) were purchased from Charles River Laboratories (Guangdong) and housed in the SPF animal research facility of Hongkong Polytechnic University, Shenzhen Research Institute. Food and water were provided *ad libitum*. All animal experiments were performed in accordance with the guidelines of the China Council on Animal Care and Use. This study was approved by the Animal Research Ethics Board of Hong Kong Polytechnic University, Shenzhen Research Institute. Every effort was made to minimize pain and discomfort to the animals.

### OVA-induced allergic rhinitis mouse model

The OVA-induced AR mouse model was established as described previously, with some modifications (38). Briefly, 36 BABL/c mice were randomly divided into a normal control group (control), model group (AR), positive control group (AZE), and strain GOLDGUT-Lpc969-treated group (GOLDGUT-Lpc969) (n=9 each group). To induce AR, mice in the AZE, AR, and GOLDGUT-Lpc969 groups were sensitized using an intraperitoneal injection of 200 μl ovalbumin (OVA) suspension (1 mg/ml OVA in saline) on days 0, 7, and 14, and then challenged daily with 500 μg OVA administered intranasally from day 21 to day 29. Mice in the control group received saline solution.

From day 14 to day 28, the probiotic intervention group received 200 μl of GOLDGUT-Lpc969 bacterial suspension at a concentration of 1×10^10^ CFU/ml in 0.9% saline by gavage daily, while the positive group received 15 mg/kg·BW AZE. Mice in the control and the model control groups received an equivalent volume of 0.9% saline. AR symptoms, including the frequency of nasal rubbing and sneezing within 10 min of intranasal administration, were recorded on day 28 and scored as 0, 1, 2, and 3 with increasing severity in all groups (39). The DAI score (**Table 1**) was calculated from the recorded scores as described previously (39). On day 29, all mice were euthanized, and samples including the blood and nasal mucosa were collected for subsequent experiments.

**Table 1:** Nasal symptom scoring in the AR mouse model.

Score	Number of nasal subbing/10 min	Number of sneezes/10 min
0	None	None
1	1–2	1–3
2	3–5	4–10
3	≥10	≥11

### Evaluation of serum IgE

The serum was separated from the collected mouse blood. A mouse IgE ELISA kit (ELISA MAX™ Deluxe Set Mouse IgE, BioLegend^®^, CA, USA) was used to quantify the serum IgE levels of the experimental mice according to the instruction manual.

The serum was first diluted twofold with Assay Buffer A. The diluted serum was added to the wells after the 96-well plate was successfully coated with the diluted capture antibody. The plate was sealed and incubated at room temperature for 2 h with shaking at 500 rpm. After washing, the reagents were added to each well in the following order: a 1-h reaction with IgE antibody, a 30-min reaction with Avidin-HRPD solution, and a 20-min reaction with 100 μl of TBM substrate in the dark. Each addition followed the procedure of sealed cultivation with shaking and four buffer washes. Finally, the stop solution was added, and the OD_450_ and OD_570_ of each well were read after 10 min of contact. The difference between OD_450_ and OD_570_ was recorded, and the IgE concentration was calculated using the standard dose-response curve.

### Serum histamine test

The level of histamine in the serum of the collected mouse blood was detected and quantified using a histamine ELISA kit (Shanghai Zhenke Biotech Co., Ltd. Shanghai, China) according to the instruction manual.

The serum was first added to a 96-well plate marked as sample wells with horseradish peroxidase (HRP)-labeled antigen, except for blank wells, and sealed for incubation at 37°C for 60 min. The plate was then washed three times with wash buffer. After the removal of residual liquid, the matrix mixture was added to all wells, and the plate was sealed for a 15-min incubation at 37°C. Finally, the stop solution was added, and the OD_450_ of each well was read after 10 min of contact. The difference between the OD_450_ of the sample wells and that of the blank group was recorded as the final absorbance value and used to calculate the histamine concentration according to the standard dose-absorbance curve generated by the histamine standard solution based on the same procedure.

### Histological analysis

The nasal mucosa of euthanized mice was fixed in 4% (m/v) paraformaldehyde. After fixation, paraffin sections were prepared and stained with hematoxylin and eosin (H&E) and periodic acid-Schiff (PAS) to observe and evaluate the histopathology of the nasal mucosa.

### Cytokine multiplex assay

The concentrations of the cytokines in the serum of the collected mouse blood were quantified using a LEGENDplex™ MU Th1/Th2 Panel (8-plex) assay kit (BioLegend^®^, CA, USA), which is a bead-based multiplex assay panel used on a flow cytometer.

The serum was first diluted twofold with assay buffer. Reagents were loaded into a V-bottom 96-well plate in the following order: assay buffer, diluted sample, and mixed beads. The plate was then sealed and incubated at 800 rpm for 2 h at room temperature. After the supernatant was discarded, the wells were washed and cleaned for antibody loading. Then, 25 µl of SA-PE was added to each well. After cultivation and cleaning, 150 μl of 1× wash buffer was added to resuspend the beads. The wells were then read by the flow cytometer, and the readings were used to calculate the cytokine concentration according to the standard dose-response curve generated by the standard solutions using the same procedure.

### Comparative genomic analysis

To further understand the mechanism underlying the efficacy of *L. paracasei* GOLDGUT-Lpc969 in AR prevention, the comparative genomic analysis was performed based on the whole-genome sequences of GOLDGUT-Lpc969 and three other *L. paracasei* strains that have been shown to be effective in alleviating respiratory disease (**Table 2**). Among them, *L. paracasei* GM-080 (28) and *L. paracasei* 362.5013889 (40) have been reported to alleviate airway disease, and *L. paracasei* strain BCRC_16100 has been reported to be ineffective in alleviating respiratory disease (28). All homologous genes within the pangenome were annotated using EggNOG v5.0.2 (41).

**Table 2:** List of *Lacticaseibacillus paracasei* strains used in the comparative genomics study.

Species	Strain	Source	Accession number	Effectiveness to airway disease
*L. paracasei* subsp. *paracasei*	GOLDGUT-Lpc969	Human feces	–	Effective (This study)
*L. paracasei*	GM-080	Human gut	NZ_CP095406.1	Effective (28)
*L. paracasei* subsp. *paracasei*	362.5013889	Human gut	NC_022112.1	Effective (40)
*L. paracasei*	BCRC-16100	Oral	NZ_CP086132.1	Non-Effective (28)
*L. paracasei* subsp. *tolerans*	FX-6-1	Kefir	NZ_CP109945.1	NA
*L. paracasei*	CLP-C10	fermented milk	NZ_CP052936.1	NA

### Statistical Analysis

Data are expressed as mean ± standard error of the mean (SEM). Differences between means were tested for statistical significance using one-way analysis of variance (ANOVA) followed by Tukeys multiple comparison test, and the homogeneity of the variance of the data was assessed using the Brown–Forsythe test and Bartletts test. Differences between groups were considered statistically significant when *p* < 0.05.

## Results

### Taxonomy and genomic analysis of *L. paracasei* GOLDGUT-Lpc969

**Figure 1:**
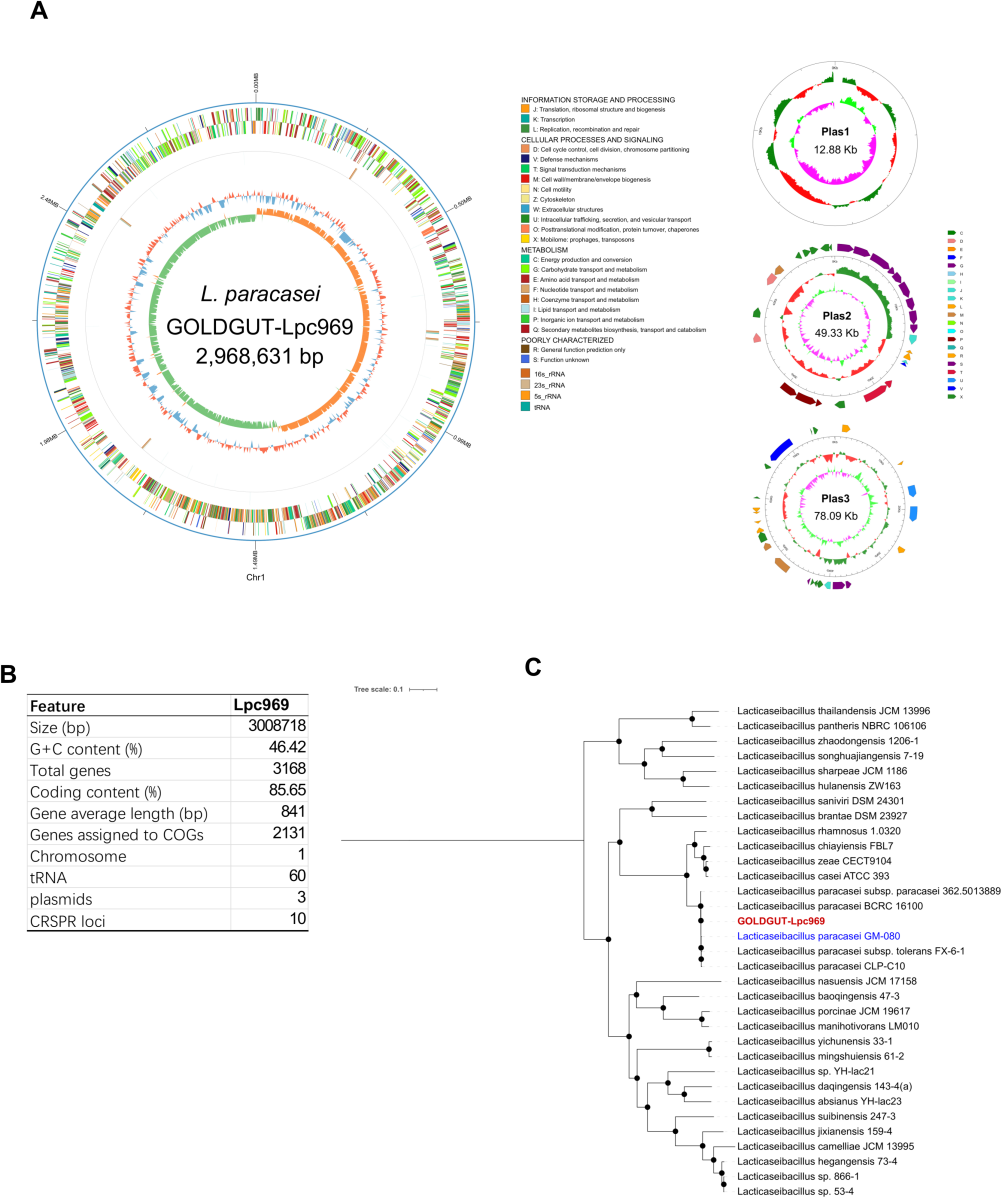
Whole-genome sequencing analysis of GOLDGUT-Lpc969.**(A)** Circular genome graph of GOLDGUT-Lpc969. **(B)** Summary of the general features of the GOLDGUT-Lpc969 genome. **(C)** Maximum likelihood tree of GOLDGUT-Lpc969 with other known *Lacticaseibacillus* strains.

Strain GOLDGUT-Lpc969 was first identified as *L. paracasei* by sequence analysis of the 16S rRNA gene using the BLAST algorithm, and the biochemical reaction detected using the API 20A strip demonstrated that strain GOLDGUT-Lpc969 can produce acid from D-glucose, D-mannitol, D-sucrose, D-mannose, D-trehalose, and D-melezitose, the unique biochemical characteristics that distinguish *L. paracasei* subsp. paracasei from *L. paracasei* subsp. *tolerans* (42). Genomic phylogenetic analysis with different *Lacticaseibacillus* species revealed that strain GOLDGUT-Lpc969 forms a distinct clade with other strains of *L. paracasei* (**Figure 1C**, **Table 2**). These results demonstrated that strain GOLDGUT-Lpc969 belongs to the species of *L. paracasei* subsp. *Paracasei*.

*L. paracasei* subsp. *paracasei* strain GOLDGUT-Lpc969 possesses a circular genome with a size of 2,968,631 base pairs (bp) and three additional circular plasmids of 12.88 kbp (kbp), 49.33 kbp, and 78.09 kbp (**Figure 1A**). A total of 3,168 protein-coding sequences assigned to 2,131 COGs were identified, and 60 tRNAs, 5 23S rRNAs, 5 16S rRNAs, 5 5S rRNAs, and 10 CRISPR sequences were predicted in the genome (**Figure 1B**).

We assessed the safety of strain GOLDGUT-Lpc969 using the whole-genome sequencing analysis standards for probiotic safety provided by the European Food Safety Authority (43). Diamond software was used to search the Virulence Factor Database to identify putative virulence factors. The sequence homologies of all these factors were less than 80%, and most of them lacked definitive functions. Subsequent analysis of the amino acid sequences encoded by the strain GOLDGUT-Lpc969 genome against the Antibiotic Resistance Genes Database revealed putative resistance genes with sequence homologies all below 55%. Taken together, these results suggest a high safety profile for strain GOLDGUT-Lpc969.

### *L. paracasei* prevents anti-DNP-IgE-induced histamine secretion and the degranulation of RBL-2H3 cells

RBL-2H3 is a basophilic leukemia cell line and is commonly used for allergy-related studies. Azelastine (AZE) is an anti-allergic reagent that inhibits the release of histamine and other inflammatory mediators. In this experiment, AZE was adopted as a positive medicine to better understand the efficacy of *L. paracasei* GOLDGUT-lpc969.

**Figure 2:**
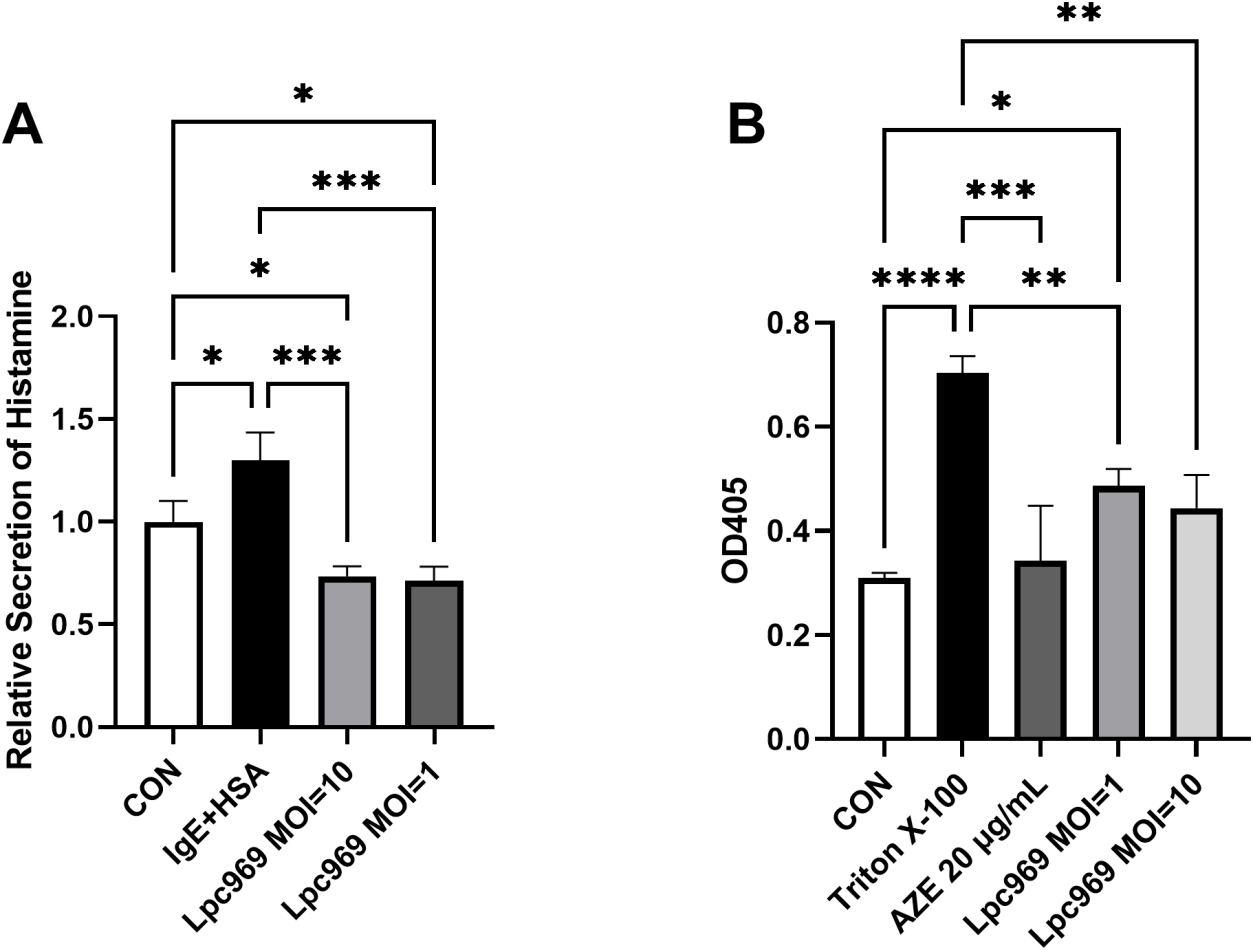
*L. paracasei* prevents anti-DNP-IgE-induced histamine secretion and the degranulation of RBL-2H3 cells. Relative secretion of histamine **(A)** and the OD405 **(B)** of the IgE+HAS treated/untreated groups. *p<0.05, **p<0.01, ***p<0.001, ****p<0.0001.

As shown in **Figure 2A**, the levels of histamine secretion by the treated RBL-2H3 cells were significantly higher than the control group (increased by approximately 63%). Although significant decreases were observed in the groups treated with *L. paracasei* GOLDGUT-Lpc969 at MOIs of 10 and 1, the significance was higher than that of AZE. These results indicate that *L. paracasei* GOLDGUT-Lpc969 can reduce histamine levels released by anti-DNP-IgE-induced RBL-2H3 cells.

The enzyme β-hexosaminidase, a marker of degranulation in RBL-2H3 cells, has been widely employed to track mast cell degranulation, as it occurs simultaneously with histamine release. The higher degranulation of RBL-2H3 cells shown in **Figure 2B** was represented by the higher OD_405_ value in a colorimetric assay. Triton X-100 is a surfactant that can cause cell rupture, resulting in cell degranulation. The stimulated RBL-2H3 cells treated with Triton X-100 showed significantly higher levels of degranulation than the control group. In contrast, significant decreases were observed in the AZE-treated groups. Although not as drastic as AZE, the intervention of *L. paracasei* GOLDGUT-Lpc969 at MOIs of 10 and 1 also significantly suppressed the degranulation level of the anti-DNP-IgE-induced RBL-2H3 model in a dose-dependent manner. These results indicate that *L. paracasei* GOLDGUT-Lpc969 can attenuate the extent of RBL-2H3 cell degranulation induced by anti-DNP-IgE.

### Oral administration of *L. paracasei* GOLDGUT-Lpc969 prevents the OVA-induced AR symptoms in mice

The evaluation of the effect of *L. paracasei* GOLDGUT-Lpc969 was assessed from four aspects: average DAI, serum IgE, serum histamine, and a histological assessment. AZE is a commonly used medicine for the topical treatment of the symptoms of AR. It acts as an immunomodulator in the anti-allergic process, serving to stabilize the mast cell membrane, thereby inhibiting the release of histamine and other inflammatory mediators from mast cells. In this experiment, AZE was adopted as a positive medicine to better understand the efficacy of *L. paracasei* GOLDGUT-Lpc969.

**Figure 3:**
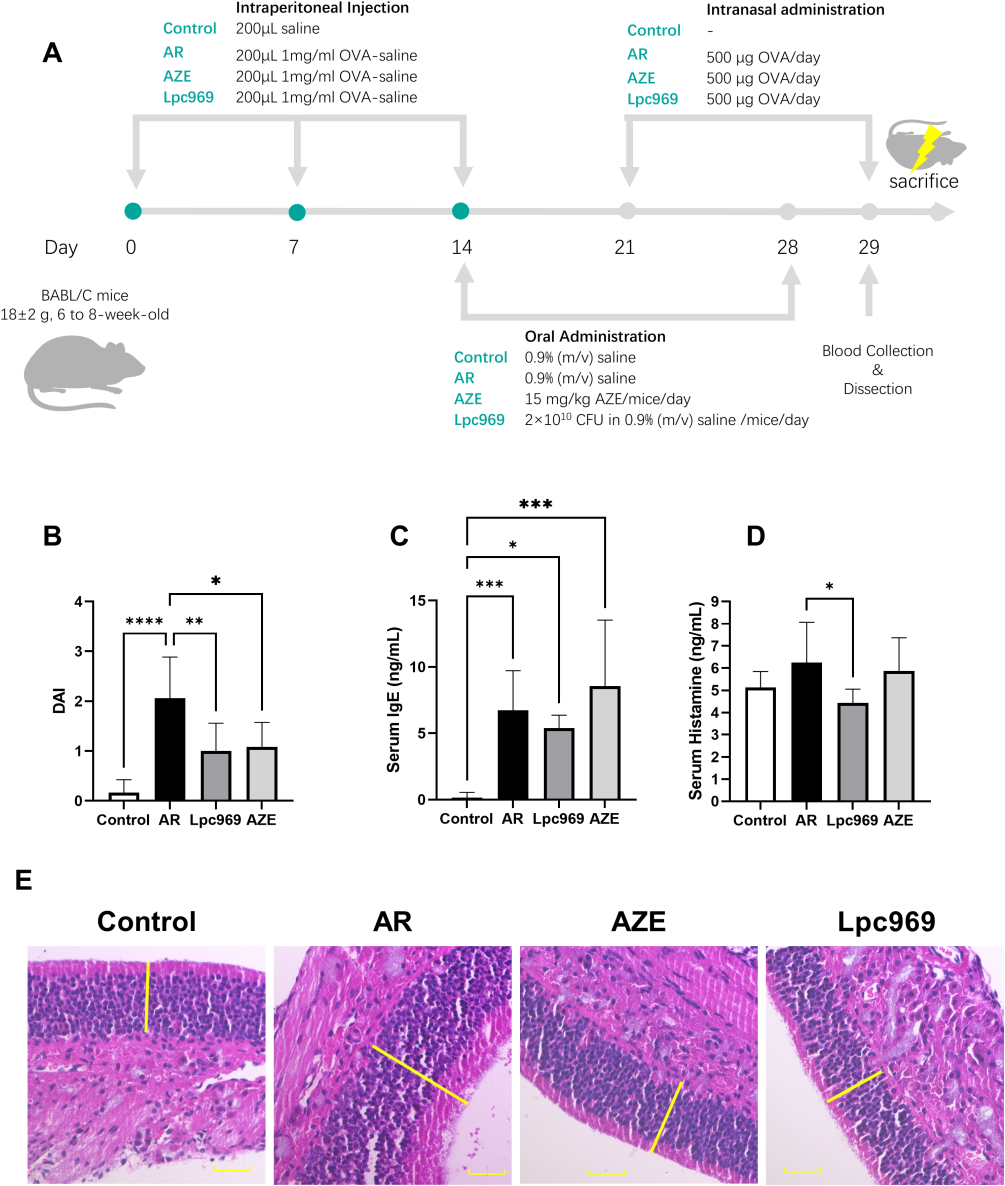
Evaluation of the protective effect of *L. paracasei* GOLDGUT-Lpc969 in OVA-induced AR mice. **(A)** Illustration of the experimental procedure used for the induction of the OVA-induced AR mouse model. **(B)** Average DAI. **(C)** Concentration of serum IgE. **(D)** Concentration of serum histamine. **(F)** Representative photomicrographs of pathological sections of the nasal mucosa of mice. *p<0.05, **p<0.01 ***p<0.001, ****p<0.0001.

The DAI score is an important indicator used to assess the symptoms of AR in mice, including scoring the frequency of sneezing, nasal rubbing, and nasal discharge. A higher score indicates more severe AR symptoms in mice, as shown in **Figure 3A**. The DAI score of mice in the AR group increased significantly compared with the control group. The score decreased significantly after the treatment with *L. paracasei* GOLDGUT-Lpc969 and AZE compared with the AR group; *L. paracasei* GOLDGUT-Lpc969 was even slower than AZE. These results demonstrated that *L. paracasei* GOLDGUT-Lpc969 can alleviate the increased DAI score caused by OVA and significantly reduce the severity of AR symptoms.

### Oral administration of *L. paracasei* GOLDGUT-Lpc969 prevents the OVA-induced elevation of serum IgE in mice

The excessive production of IgE supports the immune systems overreaction during the course of AR. Elevated blood levels of IgE are observed in most AR patients and have also been reported as a common symptom in OVA-induced AR mice. As shown in **Figure 3B**, the AR mouse group exhibited a significant increase in serum IgE after OVA sensitization compared with the control group. This phenomenon was also observed in the AZE group, which had even higher serum IgE levels than the AR group. Treatment with *L. paracasei* GOLDGUT-Lpc969 showed a decrease in serum IgE levels, indicating the potential function of the strain in preventing AR.

### Oral administration of *L. paracasei* GOLDGUT-Lpc969 prevents the OVA-induced elevation of serum histamine in mice

**Figure 4:**
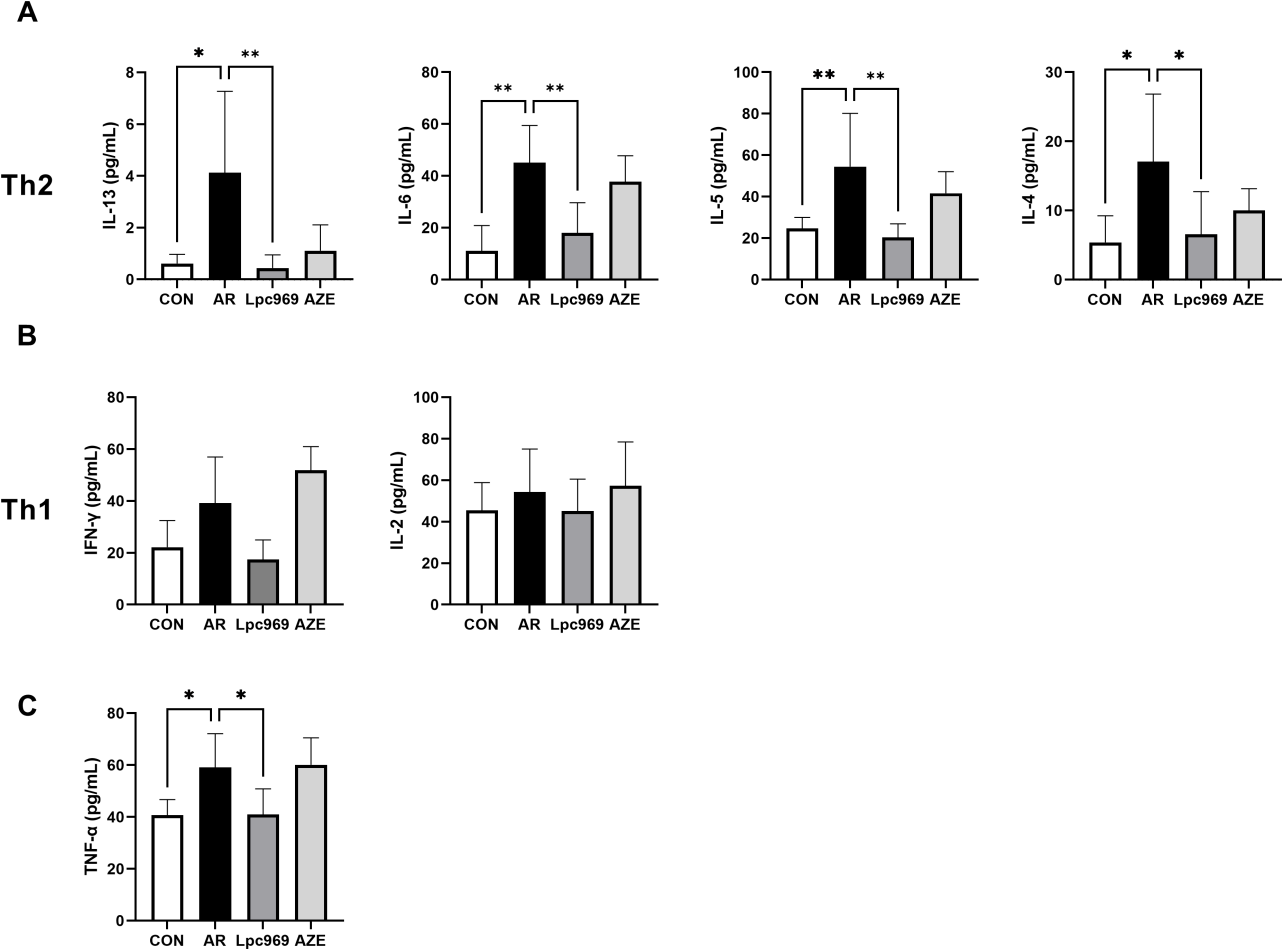
Serum cytokine levels of OVA-induced AR and treated mice. **(A)** Serum level of Th2-secreted cytokines. **(B)** Serum level of Th1-secreted cytokines. **(C)** Serum level of TNF-α. *p<0.05, **p<0.01.

Histamine is a pivotal mast-cell-producing mediator involved in local immune and inflammatory responses and is a major contributor to itching. Serum histamine levels positively correlate with the severity of allergic symptoms. As shown in **Figure 4C**, OVA-induced AR resulted in increased serum histamine levels in the AR group compared with those in the control group. This increase was slightly restored by AZE treatment, but significantly decreased by preventive treatment with *L. paracasei* GOLDGUT-Lpc969, suggesting the alleviation of histamine release and related phenomena in AR.

### Oral administration of *L. paracasei* GOLDGUT-Lpc969 prevents the OVA-induced histopathological change of nasal mucosa in mice

Compared with healthy mice, histopathologic sections of nasal mucosa from AR mice show features such as cilia loss, vascular congestion, vascular proliferation, mucosal thickening, and inflammatory cell infiltration. As shown in **Figure 3E**, mice in the control group had an intact and ordered nasal mucosal surface without mucosal enlargement. In contrast, the AR group exhibited obvious mucosal thickening, and the mucosal surface was unevenly arranged. Mice treated with *L. paracasei* GOLDGUT-Lpc969 and AZE showed obvious recovery, with intact and ordered epithelial structures and mucosal thickness restored to levels similar to those of the control group.

In conclusion, these studies demonstrated that oral administration of *L. paracasei* GOLDGUT-Lpc969 reduced the DAI in mice, along with decreased levels of IgE and histamine, and restored the nasal mucosal structure and thickness. The results support the efficacy of GOLDGUT-Lpc969 in the prevention of AR symptoms.

### Oral administration of *L. paracasei* regulates the serum cytokine level of OVA-induced murine AR

IL-13, IL-6, IL-5, and IL-4 are Th2 cytokines, whereas IFN-γ and IL-2 are Th1 cytokines. In this study, the levels of all these cytokines in mouse serum tended to increase after OVA stimulation, indicating an active Th2 and Th1 inflammatory response. AZE treatment conferred some inhibition of the secretion of Th2 cytokines, e.g., approximately 50% for IL-5 and IL4, 20% for IL-6, and 80% for IL-13, with a slight increase in the secretion of the Th1 cytokines IFN-γ and IL-2 in comparison with the AR group. *L. paracasei* GOLDGUT-Lpc969 treatment also lowered the levels of all the above cytokines close to those of the control group, showing significant suppression on the secretion of Th2 cytokines (**Figure 4A**), especially IL-13, with over a 90% reduction compared with the AR group, and a moderate inhibition on that of the Th1 cytokines (**Figure 4B**). These results suggest that *L. paracasei* GOLDGUT-Lpc969 can suppress the Th1 and Th2 inflammatory responses while alleviating AR symptoms by significantly reducing Th2 cytokines and reversing the bias of the Th2 response.

Apart from the Th1 and Th2 cytokines, the mast-cell-secreted TNF-α also significantly decreased in the probiotic group compared with the model group, recovering to a level close to that of the control group (**Figure 4C**). This result suggested a lower activity of mast cells.

### Screening of unique gene by comparison of effective/non-effective *L. paracasei* strains in treating airway disease

In a further comparative genomic analysis, we aimed to identify unique genes that were specific to the strains effective in the treatment or prevention of AR. The probiotic strain *L. paracasei* 362.5013889 was validated in a rigorous randomized placebo-controlled clinical trial, which highlighted the protective effects of *Lactiplantibacillus plantarum* HEAL9, when co-administered with *L. paracasei* 8700:2 (*L. paracasei* 362.5013889), against a range of rhinitis symptoms in adults, including nasal discharge, congestion, and sneezing (40). *L. paracasei* GM-080 has received support from studies highlighting its potential to alleviate OVA-induced airway hyperresponsiveness and reduce airway inflammation in a mouse experiment and its association with increased levels of IFN-γ in a well-controlled double-blind clinical trial. In contrast, the group treated with *L. paracasei* BCRC_16100 in the same experiment exhibited no significant increase in IFN-γ levels and no significant improvement in the other parameters assessed (28) (**Table 2**). Therefore, three effective *L. paracasei* strains, including 362.501889, GM-080, and GOLDGUT-Lpc969, in this study, and one non-effective *L. paracasei* strain, BCRC 16100, were selected for comparative genomic analysis.

**Figure 5:**
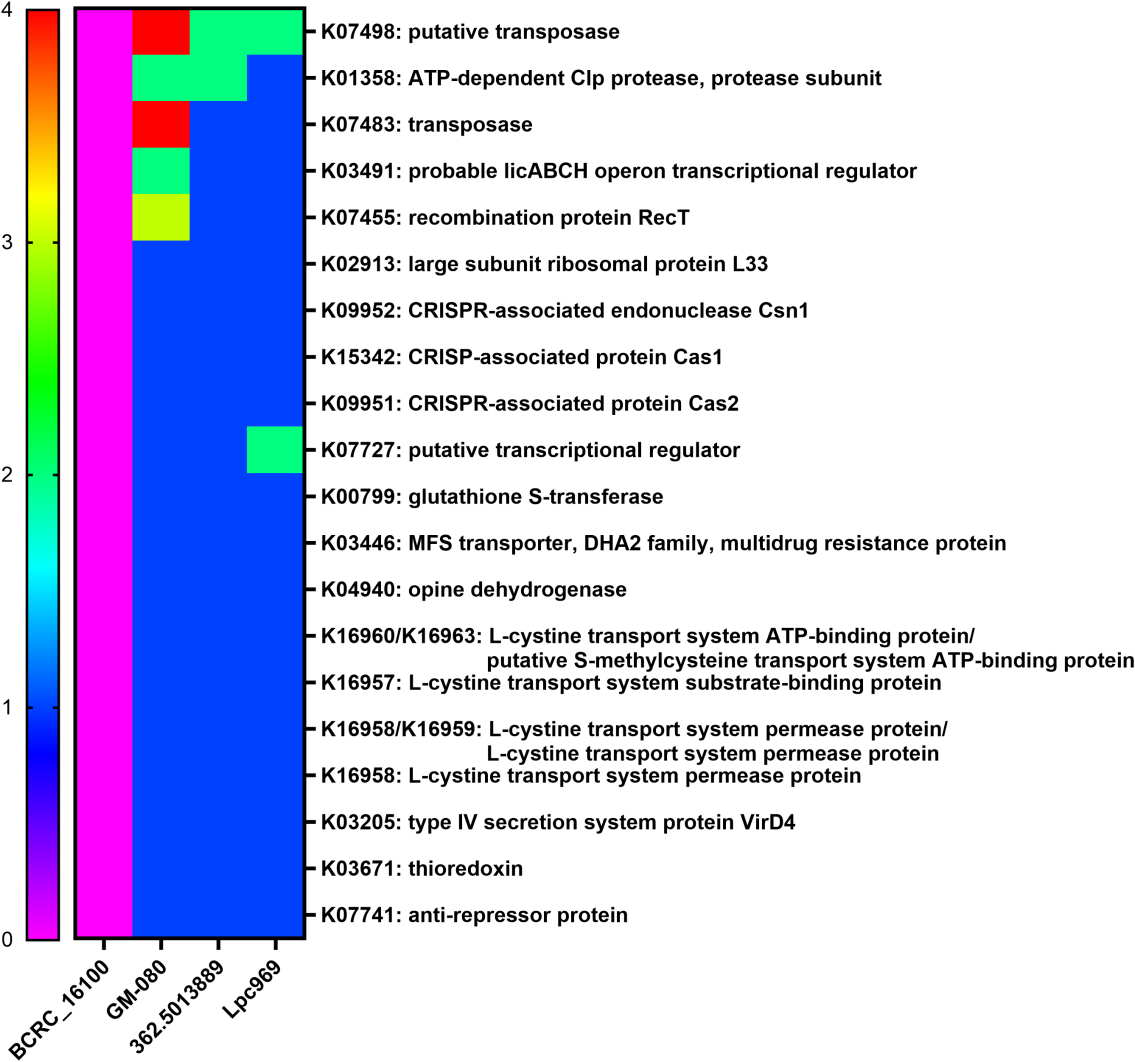
Distribution of the core genes and their copy numbers in airway-disease-effective/non-effective *L. paracasei* strains. The heatmap shows the copy number of genes solely expressed in the airway-disease-effective *L. paracasei* strains, including GOLDGUT-Lpc969, but not in the non-effective strains. Green represents the allergy-related protein.

As a result, 20 effective strain-only genes (**Figure 5**) were screened, encoding CRISPR-associated proteins, transposases, transcriptional regulators, and transport systems. Of note is the presence of K03671 (thioredoxin), a secretory protein that functions as an anti-inflammatory, an anti-allergen, a redox regulator, and an antioxidant in all effective strains. In addition, thioredoxin has been considered a promising future target for the treatment of digestive tract irritation, chronic obstructive pulmonary disease, and some allergic diseases such as allergic asthma, AR, food allergy, and contact dermatitis (44). This implies a possible correlation between the thioredoxin gene K03671 and the alleviating effect of *L. paracasei* on AR. However, empirical substantiation is required to firmly establish this association.

## Discussion

This study provides a novel strain *L. paracasei* GOLDGUT-Lpc969 with a well-characterized safety profile, determines its significant suppression on the Th2 immune response, and suggests a functional gene K03671 as a potential key gene in the prevention and alleviation of AR symptoms.

An imbalance in favor of humoral immune responses mediated by Th2 cells over cell-mediated responses led by Th1 cells has been implicated as a major cause of allergy (45). During the development of AR, the overactivation of Th2 immune responses leads to IgE-mediated type I hypersensitivity, which subsequently stimulates mast cell degranulation and histamine release (46). In turn, the histamine increases the secretion of Th2 cytokines such as IL-4, IL-5, and IL-13 and inhibits the production of the Th1 cytokines IL-2 and IFN-γ through the upregulation of prostaglandin E2 and nitric oxide (7), thereby exacerbating the allergy. The results of the present study showing a significant reduction in Th2 cytokines, serum histamine, IgE, and other disease indicators in probiotic-treated mice are consistent with the decreased histamine and degranulation rate in the *L. paracasei* GOLDGUT-Lpc969 group (**Figures 2**, **3**), indicating suppressed Th2 immune activity. Moreover, the decreased level of TNF-α suggests a reduction in mast cell activity, which contributes to the alleviation of AR symptoms (**Figure 4C**).

Based on the above mechanism, several strains have been reported and demonstrated to have protective or therapeutic effects on AR. Oral administration of *L. plantarum* NR16 isolated from kimchi was reported to ameliorate murine AR by rebalancing the Th1:Th2 ratio by decreasing Th2 cytokine production, thus inhibiting the production of allergen-specific antibodies in specific mucosal lesions (47). Similarly, 10^9^ CFU freeze-dried powder of *L. plantarum* HEAL9 and *L. paracasei* 8700:2 was clinically proven to be effective in protecting cold-prone adults from multiple colds (40). These findings justify the efficacy of specific strains in rebalancing Th1/Th2 inflammatory responses.

**Figure 6:**
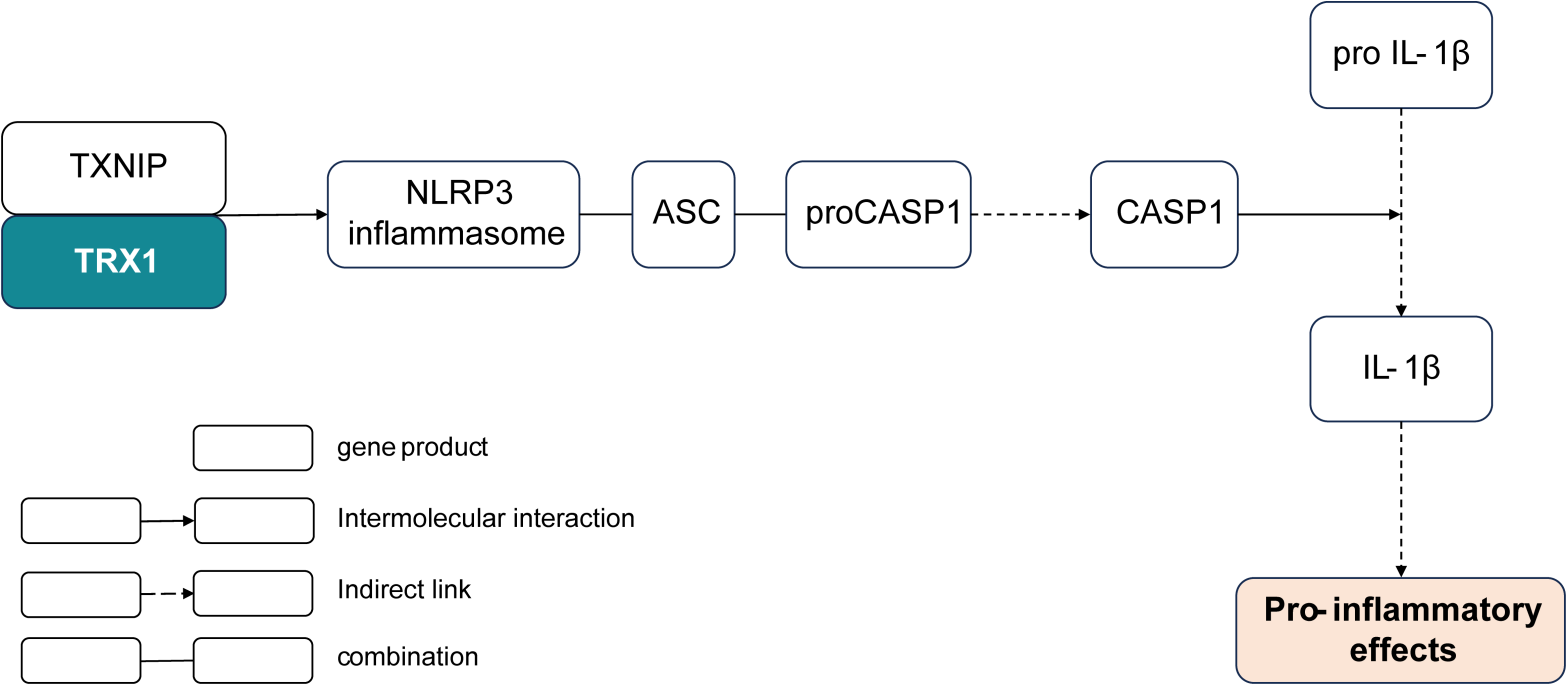
Shortcut of the K03671-encoded thioredoxin involved in the pro-inflammatory pathway. TRX, thioredoxin; TXNIP, thioredoxin-interacting protein; NLRP3, NOD-, LRR-, and pyrin domain-containing protein 3; ACS, apoptosis-associated speck-like protein containing a CARD; CASP1, caspase 1.

Subsequently, the investigation of the screened 20 effective strain-only genes revealed the potential function of the gene K03671 in alleviating respiratory allergy. The gene K03671 putatively encodes thioredoxin (TRX1), a reductase of oxidized cysteine residues and cleaver of disulfide bonds (48). In the NOD-like receptor signaling pathway, the reaction of thioredoxin and thioredoxin-interacting protein (TXNIP) is known to stimulate the expression of the NOD-, LRR-, and pyrin domain-containing protein 3 (NLRP3) inflammasome (49). Assembly of the NLRP3 inflammasome eventually leads to the caspase 1 (CASP1)-dependent release of pro-inflammatory cytokines such as interleukin-1beta (IL-1β), which can activate T cells and promote the production of Th2 cytokines, such as IL-13, IL-6, and IL-5, to stimulate pro-inflammatory effects (44, 50) (**Figure 6**). In general, the pathway determining the remarkable role of the thioredoxin encoded by K03671 in the inflammatory effects is plausibly inferred from the existing experimental and comparative genomic results, and may find further validation through additional studies.

Further research on thioredoxin revealed the mechanism of the therapeutic effect of thioredoxin on airway allergy through the downregulation of interleukin-13 (IL-13) expression and eosinophil activity. A study of the suppressive effect of thioredoxin on airway inflammation using a mouse model of asthma showed that IL-13 and exotoxin production were significantly reduced in TRX-transgenic (TRX-Tg) mice, which reduced eosinophil recruitment and decreased mucus metaplasia. This phenomenon was explained by the inhibition of the production of migration inhibitory factor, an upstream modulator of airway inflammation, in TRX-Tg mice (51). Moreover, IL-13 response status has been clinically shown to correlate with clinical responses and Th2 responses in the late phase after allergen challenge (52). These results are consistent with the significant decrease in IL-13 in *L. paracasei* GOLDGUT-Lpc969-treated AR mice compared with the model group in our study, explaining the protective effect of strain GOLDGUT-Lpc969 against AR symptoms.

In addition to the well-documented IL-13 and eosinophil activity (53, 54), research has also indicated the regulation of thioredoxin, including the reduction of goblet cell proliferation (55), eosinophil infiltration (56), the viscosity of cystic fibrosis sputum (57), and leukocyte infiltration into sites of inflammation (58), which contributes to the allergic symptoms. However, there is a study that suggests that intra-thioredoxin treatment exerts no obvious influence on systemic Th1/Th2 immune modulation (51), which is inconsistent with the experimental results obtained from the significant suppression of the Th2 immune response of *L. paracasei* GOLDGUT-Lpc969 presented in this study. Furthermore, a study suggests that the downregulation of SHP-1 leads to a shift toward a Th2-type immunity (59). On the one hand, TRX can reactivate SHP-2 to a catalytic structure by reduction (60), whereas thioredoxin reductase (TRR) can maintain the reduction in the oxidized TRX. SHP-1, which shares structural similarities with SHP-2, acts as a negative regulator of cell proliferation, migration, and survival through dephosphorylation, whereas SHP-2 acts as a positive factor through phosphorylation (60–63). Functionally, they exhibit antagonistic effects, potentially neutralizing or balancing each others activities. Therefore, it is plausible to infer that thioredoxin expressed by K03671 influences the activity of SHP-2 through the TRX/TRR system, thereby downregulating the expression or activity of SHP-1 to some certain extent and indirectly promoting the upregulation of Th2 cytokines, which is opposite to the experimental results. As a result, it is inferred that there is an uncovered relationship between SHP-1 and the TRX/TRR/TRXIP system. Conversely, thioredoxin (TRX) can inhibit the formation of hydrogen peroxide and free radicals (64) and prevent the increased Th2 immune response induced by H_2_O_2_ (65). Accordingly, it provides insight into the preference for further exploration focusing on the gut-lung axis to determine the function of effective genes in probiotics, especially *L. paracasei*, in the oxidative microenvironment of respiratory health.

Our investigation of the therapeutic strain effective against AR has revealed the potential key gene K03671 in the amelioration of AR symptoms, contributing to our understanding of the underlying mechanism of AR-effective *L. paracasei* in ways that were previously unexplored. However, several intriguing issues remain unanswered, such as the verification of the thioredoxin production of the AR-effective strains and the significantly reduced Th2 activity. Addressing these gaps will undoubtedly deepen our understanding of the screening strategy for AR-effective strains. Subsequent research efforts could focus on the development of other effective genes in AR-effective strains and the determination of K03671 expression.

## Data Availability

The data presented in the study are deposited in the NCBI (National Center for Biotechnology Information) repository, accession number SRX24771855.
